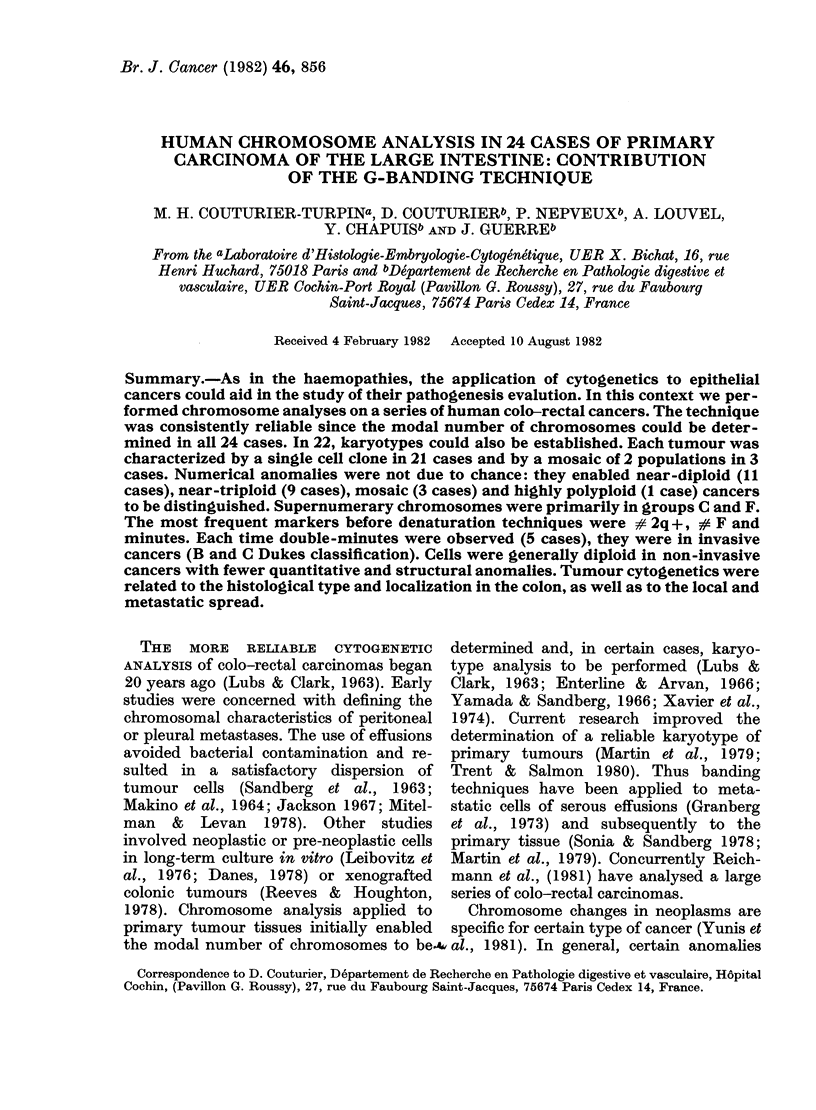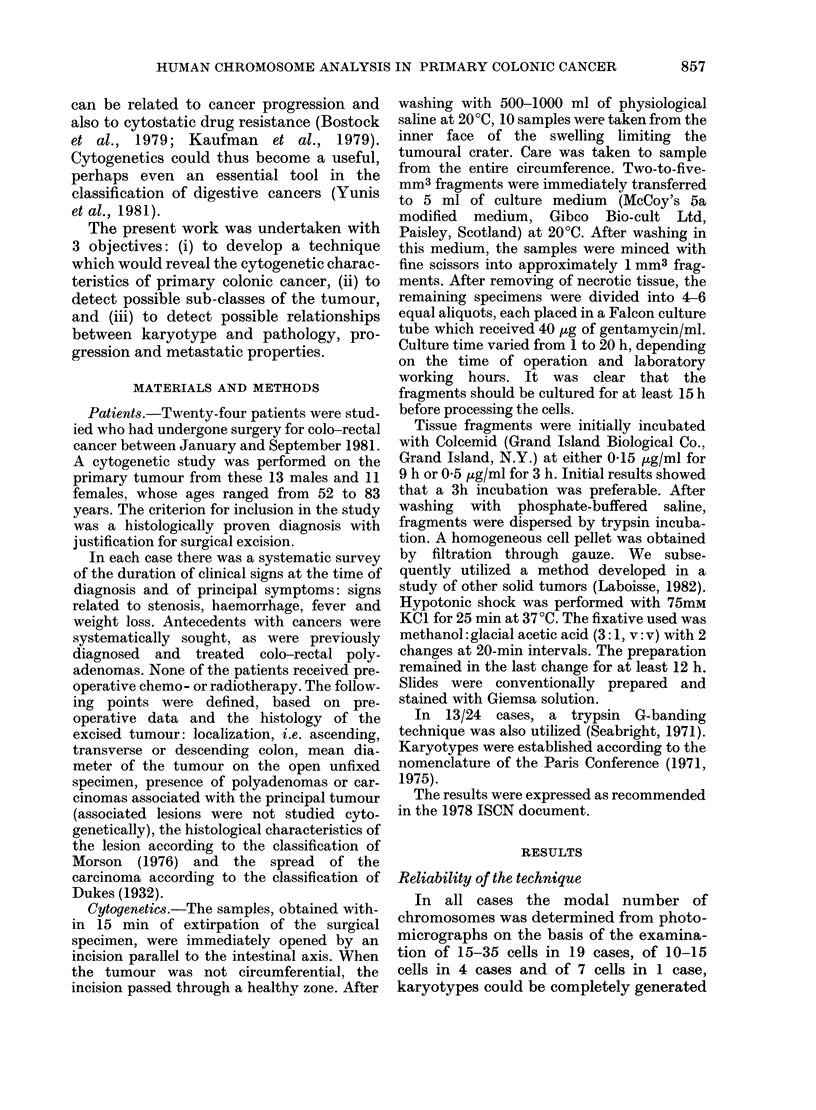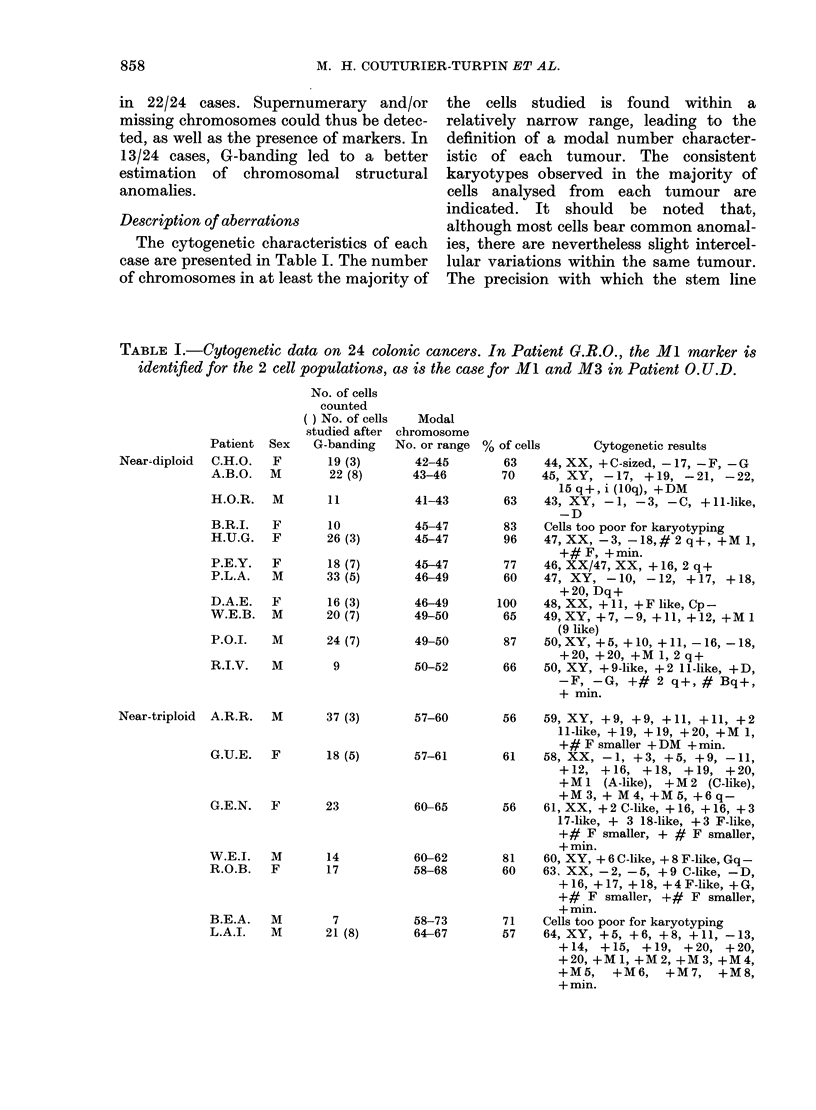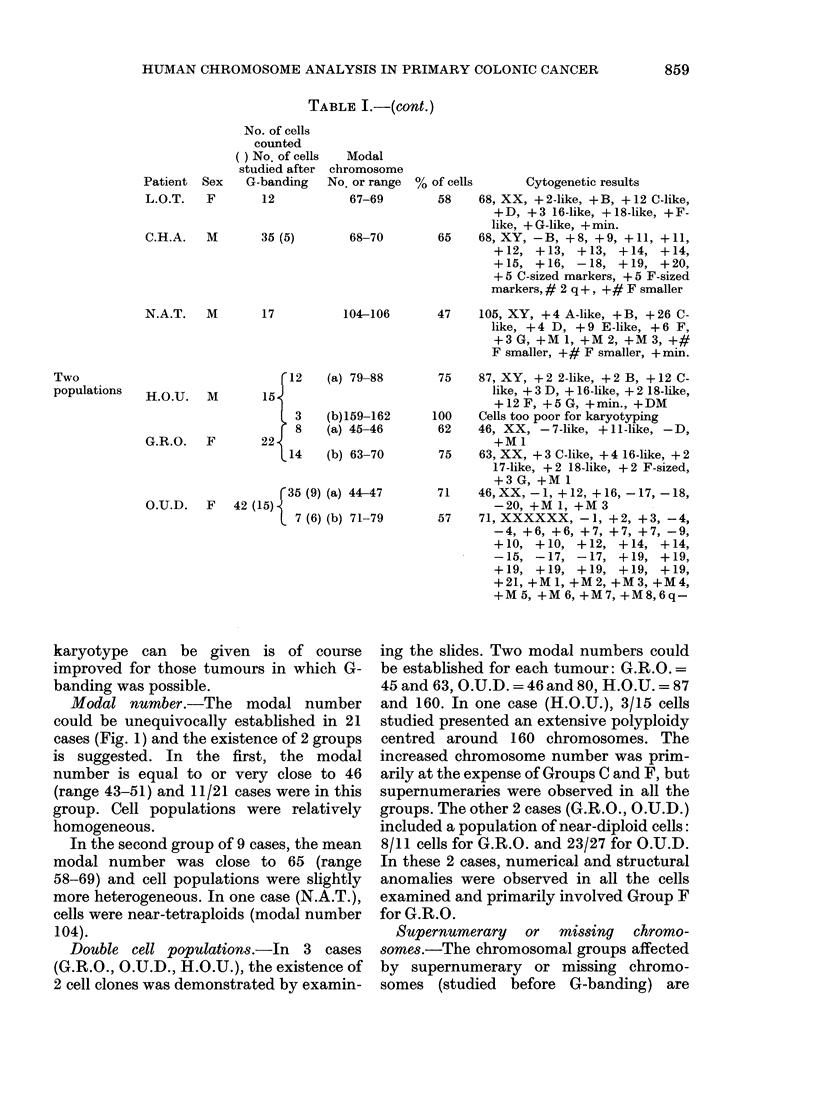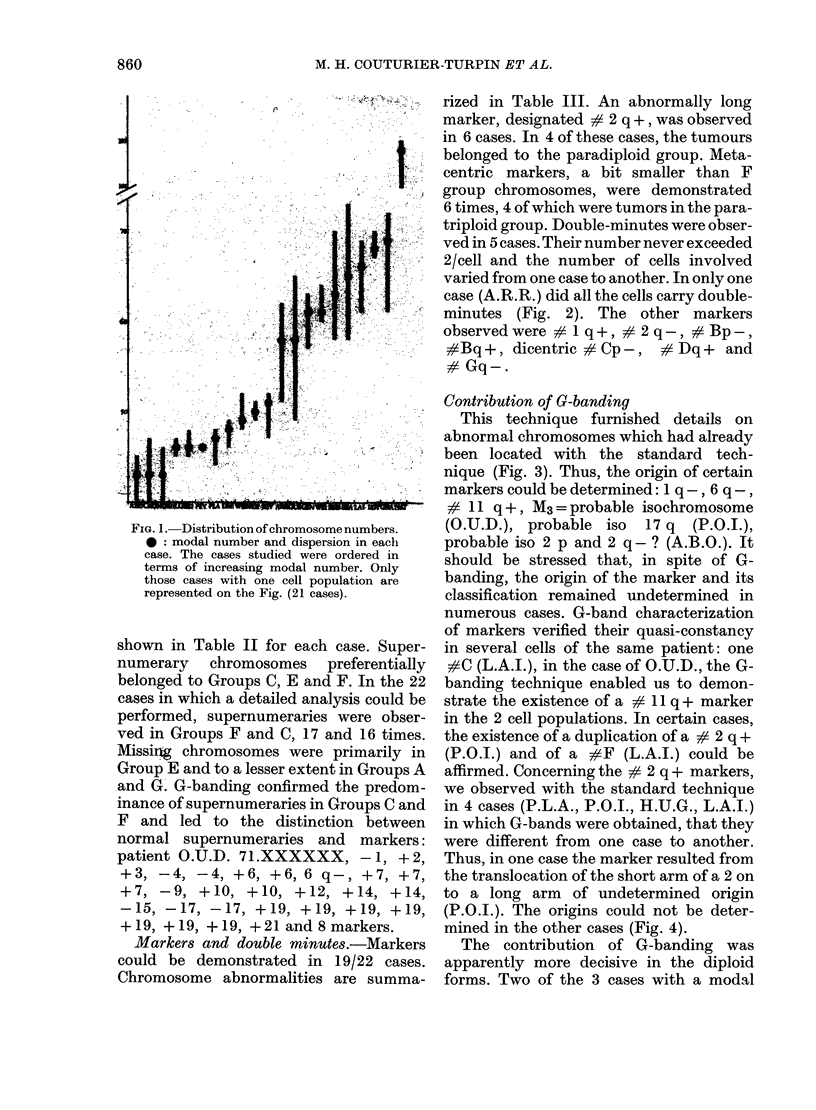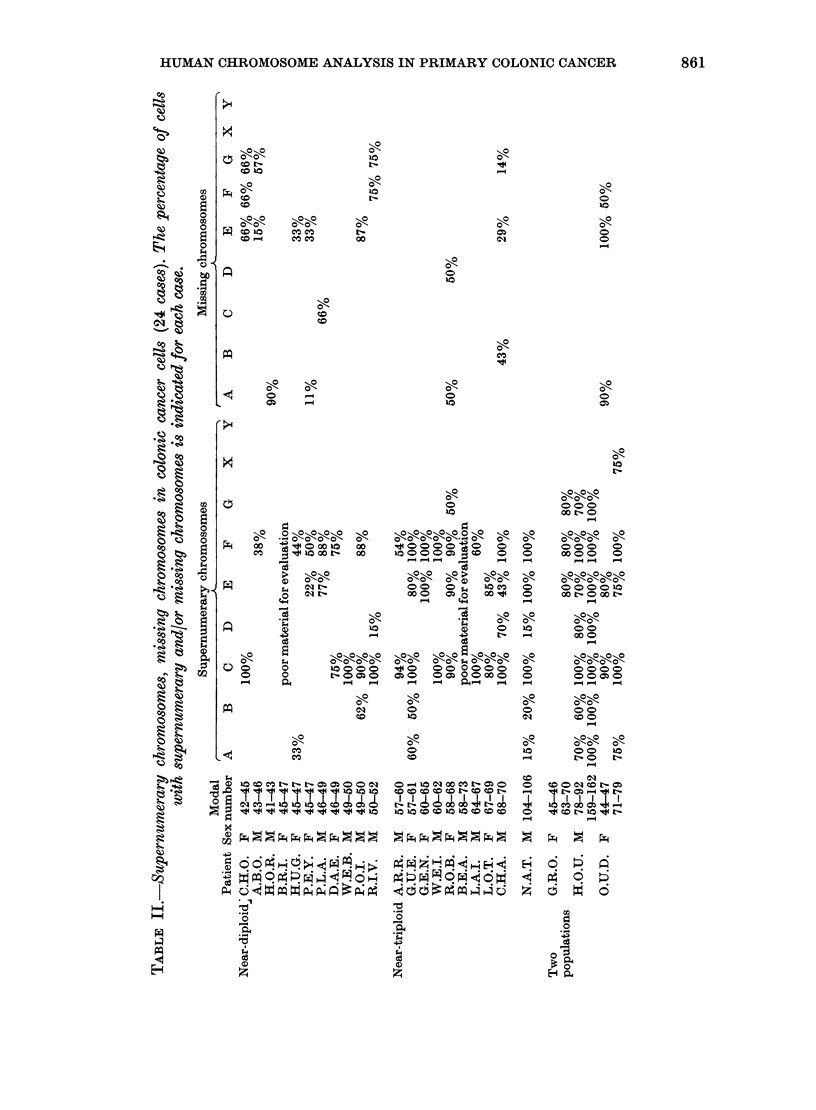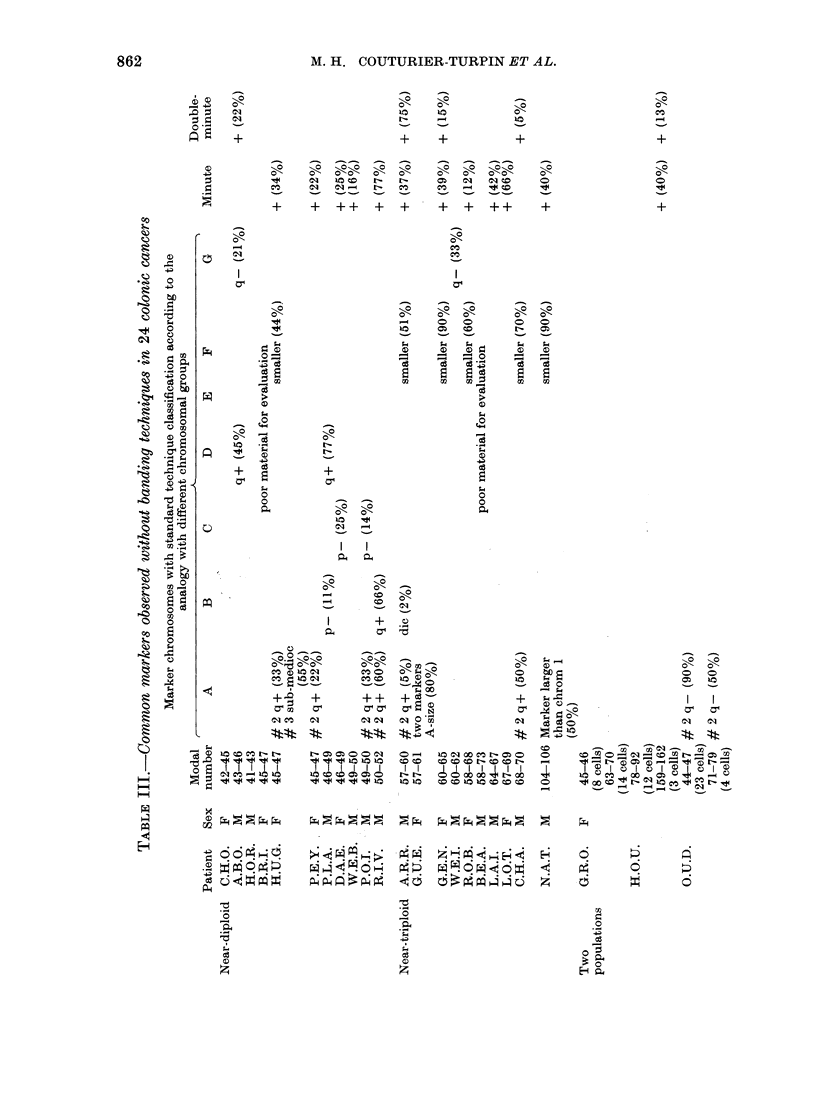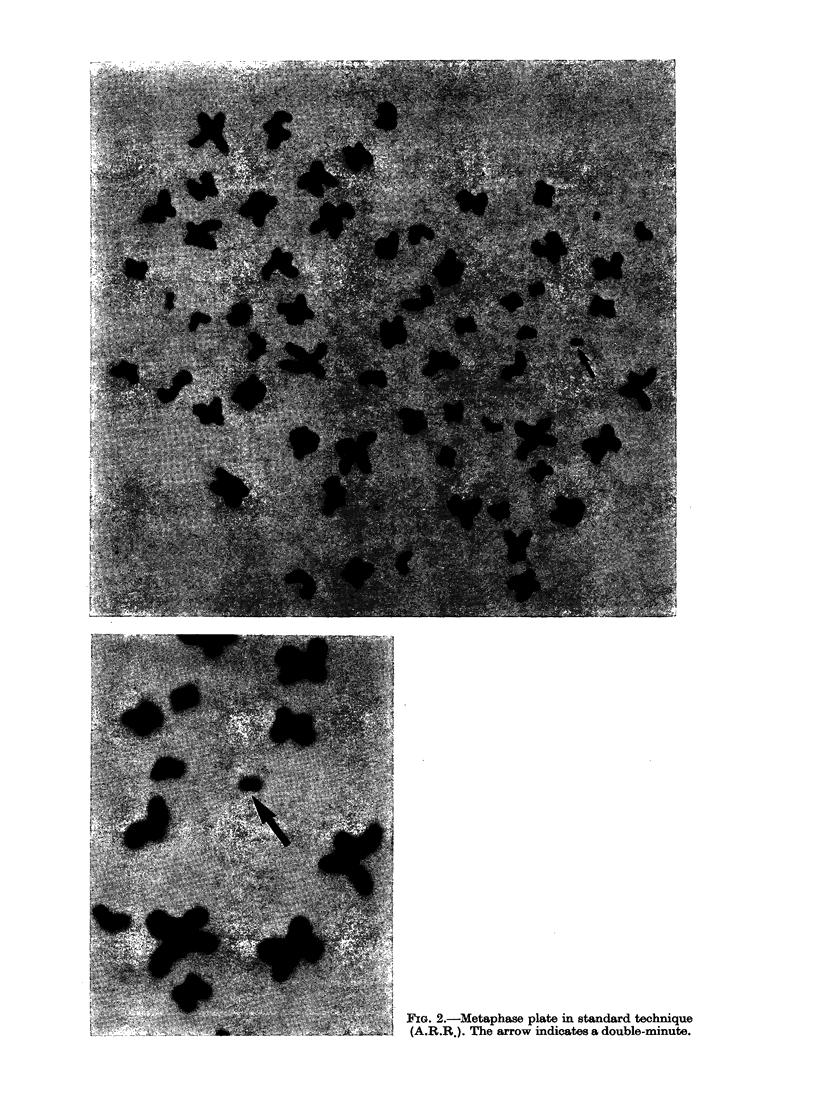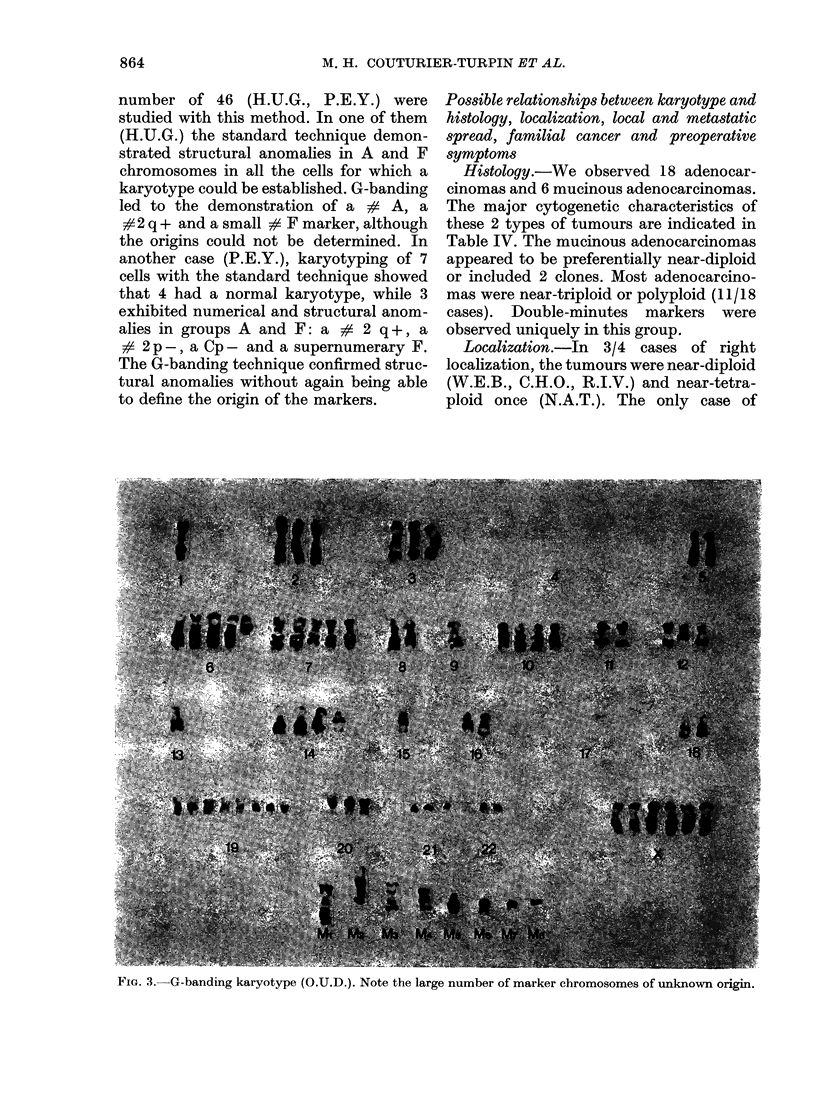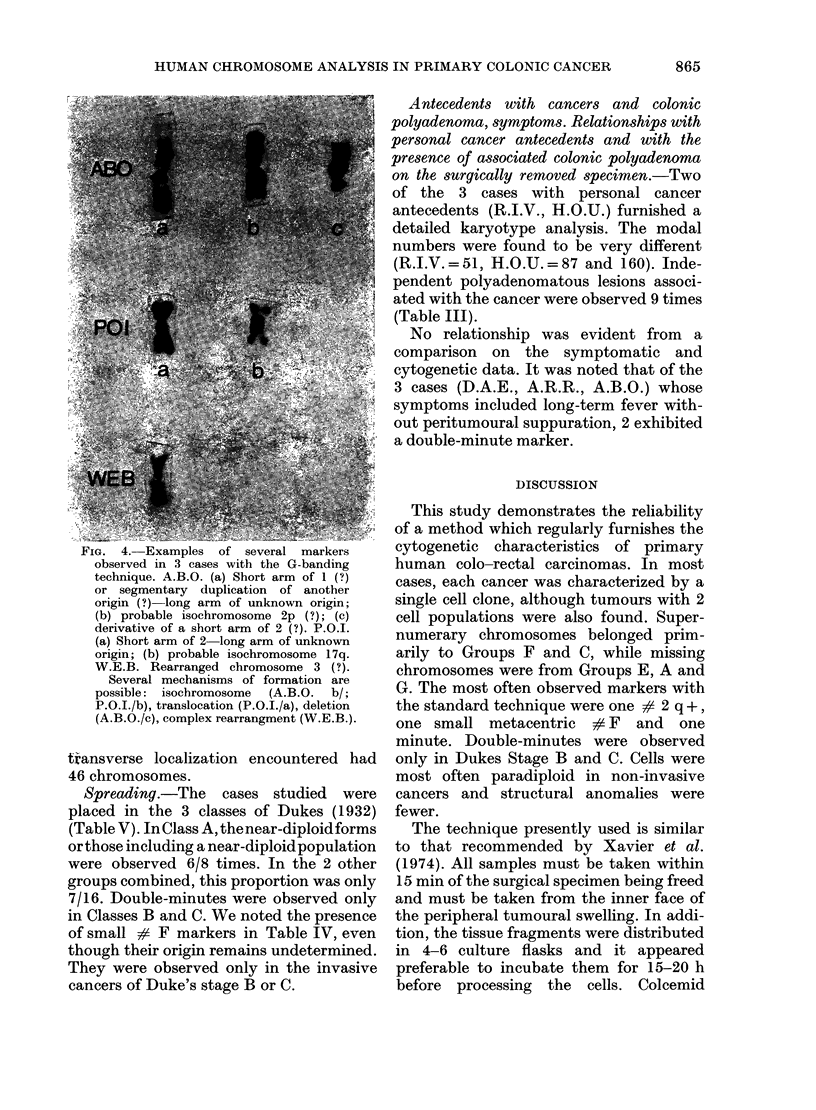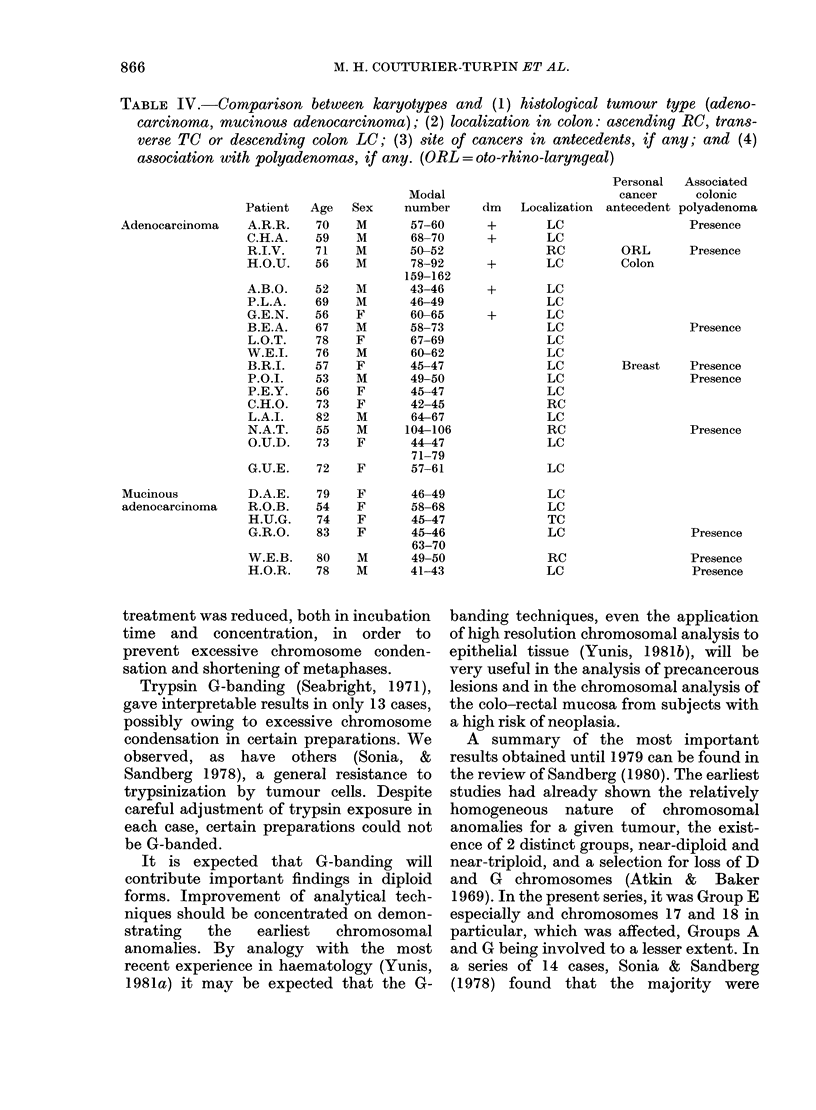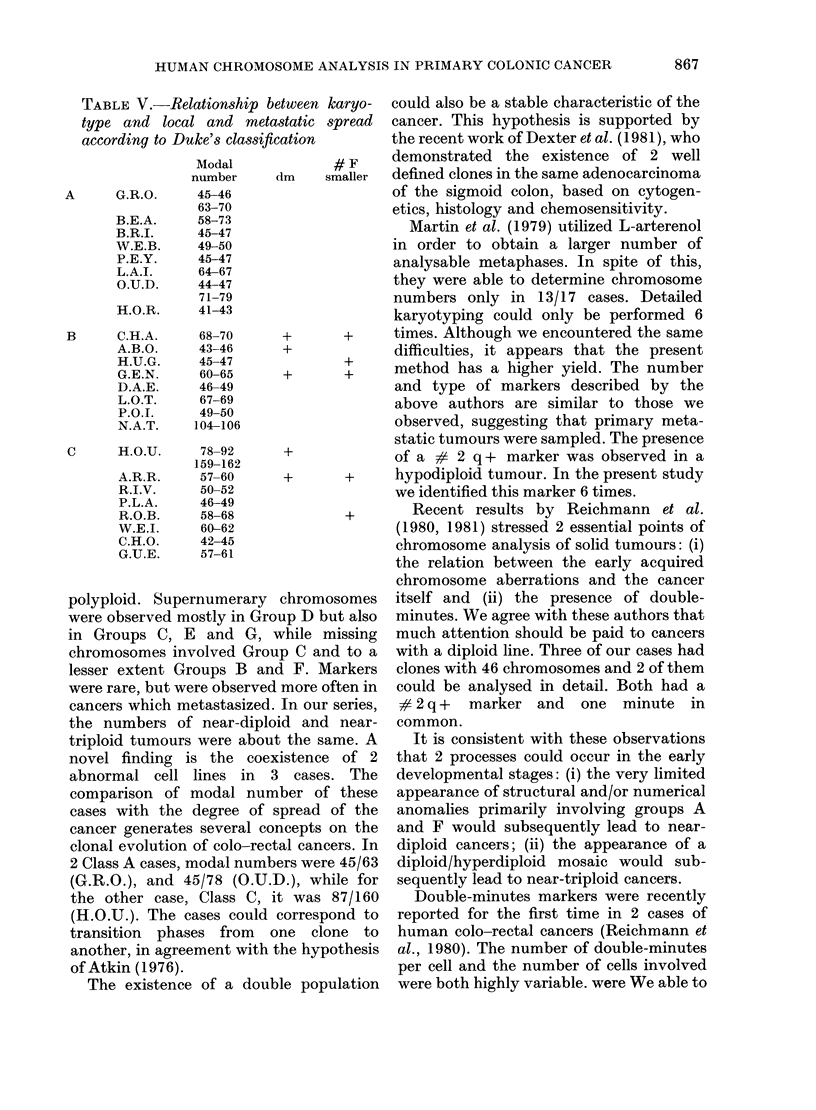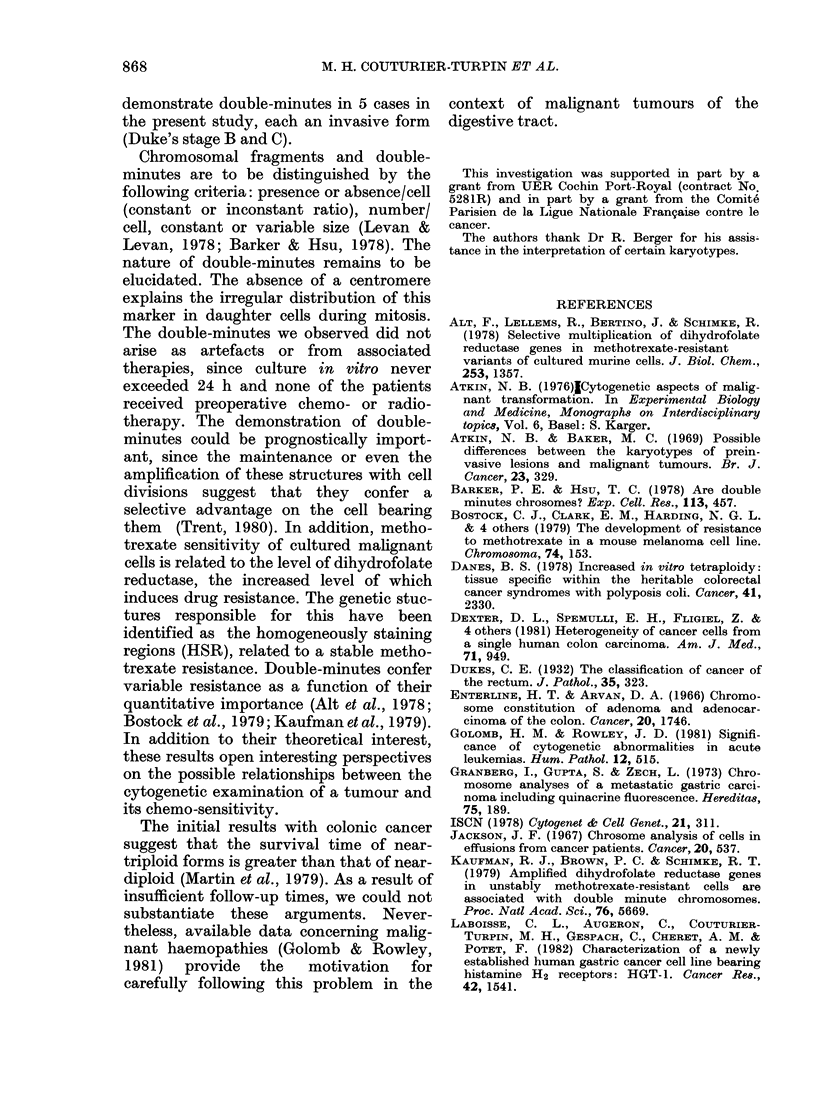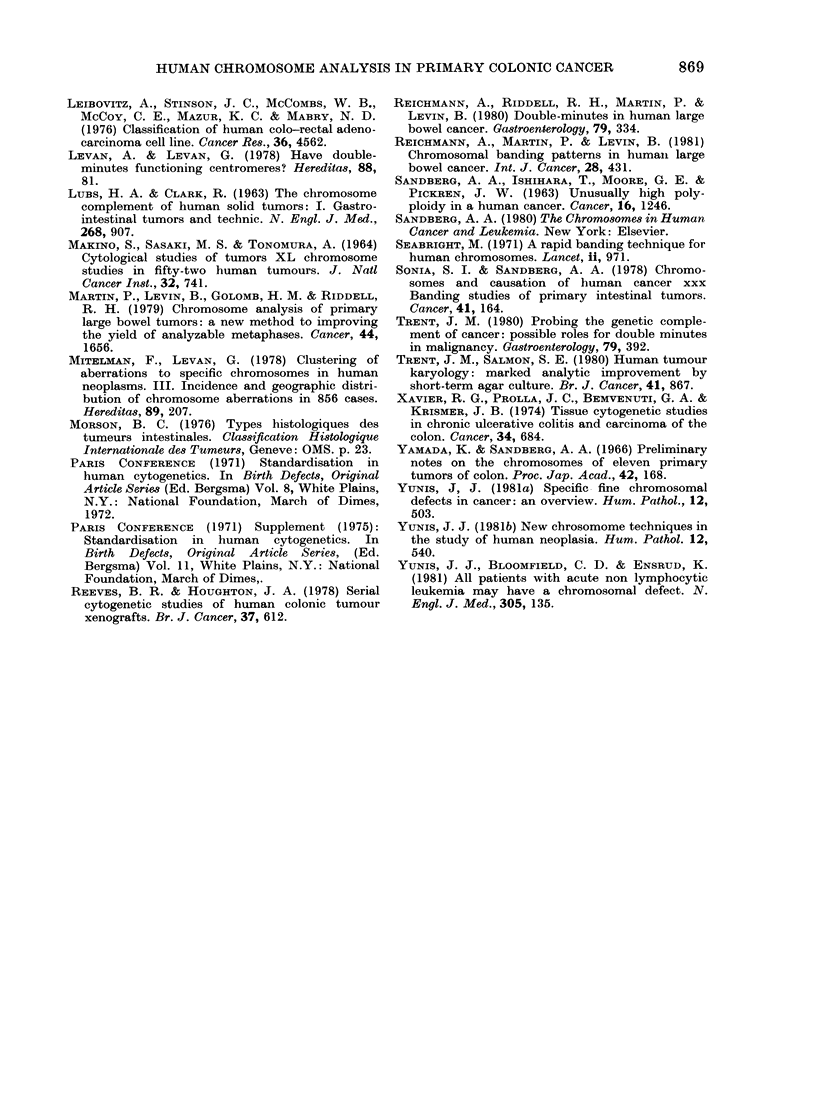# Human chromosome analysis in 24 cases of primary carcinoma of the large intestine: contribution of the G-banding technique.

**DOI:** 10.1038/bjc.1982.295

**Published:** 1982-12

**Authors:** M. H. Couturier-Turpin, D. Couturier, P. Nepveux, A. Louvel, Y. Chapuis, J. Guerre

## Abstract

**Images:**


*Br. J. Cancer* (1982) **46**, 856

### HUMAN CHROMOSOME ANALYSIS IN 24 CASES OF PRIMARY CARCINOMA OF THE LARGE INTESTINE: CONTRIBUTION OF THE G-BANDING TECHNIQUE

M. H. COUTURIER-TURPIN[a], D. COUTURIER[b], P. NEPVEUX[b], A. LOUVEL, Y. CHAPUIS[b] AND J. GUERRE[b]

*From the [a]Laboratoire d'Histologie-Embryologie-Cytogénétique, UER X. Bichat, 16, rue Henri Huchard, 75018 Paris and [b]Département de Recherche en Pathologie digestive et vasculaire, UER Cochin-Port Royal (Pavillon G. Roussy), 27, rue du Faubourg Saint-Jacques, 75674 Paris Cedex 14, France*

Received 4 February 1982 Accepted 10 August 1982

**Summary.—As in the haemopathies, the application of cytogenetics to epithelial cancers could aid in the study of their pathogenesis evalution. In this context we performed chromosome analyses on a series of human colo–rectal cancers. The technique was consistently reliable since the modal number of chromosomes could be determined in all 24 cases. In 22, karyotypes could also be established. Each tumour was characterized by a single cell clone in 21 cases and by a mosaic of 2 populations in 3 cases. Numerical anomalies were not due to chance: they enabled near-diploid (11 cases), near-triploid (9 cases), mosaic (3 cases) and highly polyploid (1 case) cancers to be distinguished. Supernumerary chromosomes were primarily in groups C and F. The most frequent markers before denaturation techniques were # 2q+, # F and minutes. Each time double-minutes were observed (5 cases), they were in invasive cancers (B and C Dukes classification). Cells were generally diploid in non-invasive cancers with fewer quantitative and structural anomalies. Tumour cytogenetics were related to the histological type and localization in the colon, as well as to the local and metastatic spread.**

THE MORE RELIABLE CYTOGENETIC ANALYSIS of colo–rectal carcinomas began 20 years ago (Lubs & Clark, 1963). Early studies were concerned with defining the chromosomal characteristics of peritoneal or pleural metastases. The use of effusions avoided bacterial contamination and resulted in a satisfactory dispersion of tumour cells (Sandberg *et al.*, 1963; Makino *et al.*, 1964; Jackson 1967; Mitelman & Levan 1978). Other studies involved neoplastic or pre-neoplastic cells in long-term culture *in vitro* (Leibovitz *et al.*, 1976; Danes, 1978) or xenografted colonic tumours (Reeves & Houghton, 1978). Chromosome analysis applied to primary tumour tissues initially enabled the modal number of chromosomes to be determined and, in certain cases, karyotype analysis to be performed (Lubs & Clark, 1963; Enterline & Arvan, 1966; Yamada & Sandberg, 1966; Xavier *et al.*, 1974). Current research improved the determination of a reliable karyotype of primary tumours (Martin *et al.*, 1979; Trent & Salmon 1980). Thus banding techniques have been applied to metastatic cells of serous effusions (Granberg *et al.*, 1973) and subsequently to the primary tissue (Sonia & Sandberg 1978; Martin *et al.*, 1979). Concurrently Reichmann *et al.*, (1981) have analysed a large series of colo–rectal carcinomas.

Chromosome changes in neoplasms are specific for certain type of cancer (Yunis *et al.*, 1981). In general, certain anomalies

Correspondence to D. Couturier, Département de Recherche en Pathologie digestive et vasculaire, Hôpital Cochin, (Pavillon G. Roussy), 27, rue du Faubourg Saint-Jacques, 75674 Paris Cedex 14, France.

can be related to cancer progression and also to cytostatic drug resistance (Bostock *et al.*, 1979; Kaufman *et al.*, 1979). Cytogenetics could thus become a useful, perhaps even an essential tool in the classification of digestive cancers (Yunis *et al.*, 1981).

The present work was undertaken with 3 objectives: (i) to develop a technique which would reveal the cytogenetic characteristics of primary colonic cancer, (ii) to detect possible sub-classes of the tumour, and (iii) to detect possible relationships between karyotype and pathology, progression and metastatic properties.

### MATERIALS AND METHODS

*Patients.*—Twenty-four patients were studied who had undergone surgery for colo–rectal cancer between January and September 1981. A cytogenetic study was performed on the primary tumour from these 13 males and 11 females, whose ages ranged from 52 to 83 years. The criterion for inclusion in the study was a histologically proven diagnosis with justification for surgical excision.

In each case there was a systematic survey of the duration of clinical signs at the time of diagnosis and of principal symptoms: signs related to stenosis, haemorrhage, fever and weight loss. Antecedents with cancers were systematically sought, as were previously diagnosed and treated colo–rectal polyadenomas. None of the patients received preoperative chemo- or radiotherapy. The following points were defined, based on preoperative data and the histology of the excised tumour: localization, *i.e.* ascending, transverse or descending colon, mean diameter of the tumour on the open unfixed specimen, presence of polyadenomas or carcinomas associated with the principal tumour (associated lesions were not studied cytogenetically), the histological characteristics of the lesion according to the classification of Morson (1976) and the spread of the carcinoma according to the classification of Dukes (1932).

*Cytogenetics.*—The samples, obtained within 15 min of extirpation of the surgical specimen, were immediately opened by an incision parallel to the intestinal axis. When the tumour was not circumferential, the incision passed through a healthy zone. After washing with 500–1000 ml of physiological saline at 20 °C, 10 samples were taken from the inner face of the swelling limiting the tumoural crater. Care was taken to sample from the entire circumference. Two-to-five-$mm^3$ fragments were immediately transferred to 5 ml of culture medium (McCoy's 5a modified medium, Gibco Bio-cult Ltd, Paisley, Scotland) at 20 °C. After washing in this medium, the samples were minced with fine scissors into approximately 1 $mm^3$ fragments. After removing of necrotic tissue, the remaining specimens were divided into 4–6 equal aliquots, each placed in a Falcon culture tube which received 40 $\mu$g of gentamycin/ml. Culture time varied from 1 to 20 h, depending on the time of operation and laboratory working hours. It was clear that the fragments should be cultured for at least 15 h before processing the cells.

Tissue fragments were initially incubated with Colcemid (Grand Island Biological Co., Grand Island, N.Y.) at either 0·15 $\mu$g/ml for 9 h or 0·5 $\mu$g/ml for 3 h. Initial results showed that a 3h incubation was preferable. After washing with phosphate-buffered saline, fragments were dispersed by trypsin incubation. A homogeneous cell pellet was obtained by filtration through gauze. We subsequently utilized a method developed in a study of other solid tumors (Laboisse, 1982). Hypotonic shock was performed with 75mM KCl for 25 min at 37 °C. The fixative used was methanol:glacial acetic acid (3:1, v:v) with 2 changes at 20-min intervals. The preparation remained in the last change for at least 12 h. Slides were conventionally prepared and stained with Giemsa solution.

In 13/24 cases, a trypsin G-banding technique was also utilized (Seabright, 1971). Karyotypes were established according to the nomenclature of the Paris Conference (1971, 1975).

The results were expressed as recommended in the 1978 ISCN document.

### RESULTS

### *Reliability of the technique*

In all cases the modal number of chromosomes was determined from photomicrographs on the basis of the examination of 15–35 cells in 19 cases, of 10–15 cells in 4 cases and of 7 cells in 1 case, karyotypes could be completely generated in 22/24 cases. Supernumerary and/or missing chromosomes could thus be detected, as well as the presence of markers. In 13/24 cases, G-banding led to a better estimation of chromosomal structural anomalies.

*Description of aberrations*

The cytogenetic characteristics of each case are presented in Table I. The number of chromosomes in at least the majority of the cells studied is found within a relatively narrow range, leading to the definition of a modal number characteristic of each tumour. The consistent karyotypes observed in the majority of cells analysed from each tumour are indicated. It should be noted that, although most cells bear common anomalies, there are nevertheless slight intercellular variations within the same tumour. The precision with which the stem line

TABLE I.—*Cytogenetic data on 24 colonic cancers. In Patient G.R.O., the M*1 *marker is identified for the* 2 *cell populations, as is the case for M*1 *and M*3 *in Patient O.U.D.*

| | Patient | Sex | No. of cells counted ( ) No. of cells studied after G-banding | Modal chromosome No. or range | % of cells | Cytogenetic results |
|---|---|---|---|---|---|---|
| Near-diploid | C.H.O. | F | 19 (3) | 42–45 | 63 | 44, XX, +C-sized, −17, −F, −G |
| | A.B.O. | M | 22 (8) | 43–46 | 70 | 45, XY, −17, +19, −21, −22, 15 q+, i (10q), +DM |
| | H.O.R. | M | 11 | 41–43 | 63 | 43, XY, −1, −3, −C, +11-like, −D |
| | B.R.I. | F | 10 | 45–47 | 83 | Cells too poor for karyotyping |
| | H.U.G. | F | 26 (3) | 45–47 | 96 | 47, XX, −3, −18, # 2 q+, +M 1, +# F, +min. |
| | P.E.Y. | F | 18 (7) | 45–47 | 77 | 46, XX/47, XX, +16, 2 q+ |
| | P.L.A. | M | 33 (5) | 46–49 | 60 | 47, XY, −10, −12, +17, +18, +20, Dq+ |
| | D.A.E. | F | 16 (3) | 46–49 | 100 | 48, XX, +11, +F like, Cp− |
| | W.E.B. | M | 20 (7) | 49–50 | 65 | 49, XY, +7, −9, +11, +12, +M 1 (9 like) |
| | P.O.I. | M | 24 (7) | 49–50 | 87 | 50, XY, +5, +10, +11, −16, −18, +20, +20, +M 1, 2 q+ |
| | R.I.V. | M | 9 | 50–52 | 66 | 50, XY, +9-like, +2 11-like, +D, −F, −G, +# 2 q+, # Bq+, + min. |
| Near-triploid | A.R.R. | M | 37 (3) | 57–60 | 56 | 59, XY, +9, +9, +11, +11, +2 11-like, +19, +19, +20, +M 1, +# F smaller +DM +min. |
| | G.U.E. | F | 18 (5) | 57–61 | 61 | 58, XX, −1, +3, +5, +9, −11, +12, +16, +18, +19, +20, +M 1 (A-like), +M 2 (C-like), +M 3, + M 4, +M 5, +6 q− |
| | G.E.N. | F | 23 | 60–65 | 56 | 61, XX, +2 C-like, +16, +16, +3 17-like, + 3 18-like, +3 F-like, +# F smaller, + # F smaller, +min. |
| | W.E.I. | M | 14 | 60–62 | 81 | 60, XY, +6 C-like, +8 F-like, Gq− |
| | R.O.B. | F | 17 | 58–68 | 60 | 63, XX, −2, −5, +9 C-like, −D, +16, +17, +18, +4 F-like, +G, +# F smaller, +# F smaller, +min. |
| | B.E.A. | M | 7 | 58–73 | 71 | Cells too poor for karyotyping |
| | L.A.I. | M | 21 (8) | 64–67 | 57 | 64, XY, +5, +6, +8, +11, −13, +14, +15, +19, +20, +20, +20, +M 1, +M 2, +M 3, +M 4, +M 5, +M 6, +M 7, +M 8, +min. |

TABLE I.—(*cont.*)

| | Patient | Sex | No. of cells counted ( ) No. of cells studied after G-banding | Modal chromosome No. or range | % of cells | Cytogenetic results |
|---|---|---|---|---|---|---|
| | L.O.T. | F | 12 | 67–69 | 58 | 68, XX, +2-like, +B, +12 C-like, +D, +3 16-like, +18-like, +F-like, +G-like, +min. |
| | C.H.A. | M | 35 (5) | 68–70 | 65 | 68, XY, −B, +8, +9, +11, +11, +12, +13, +13, +14, +14, +15, +16, −18, +19, +20, +5 C-sized markers, +5 F-sized markers, # 2 q+, +# F smaller |
| | N.A.T. | M | 17 | 104–106 | 47 | 105, XY, +4 A-like, +B, +26 C-like, +4 D, +9 E-like, +6 F, +3 G, +M 1, +M 2, +M 3, +# F smaller, +# F smaller, +min. |
| Two populations | H.O.U. | M | 15 { 12 | (a) 79–88 | 75 | 87, XY, +2 2-like, +2 B, +12 C-like, +3 D, +16-like, +2 18-like, +12 F, +5 G, +min., +DM |
| | | | 3 | (b) 159–162 | 100 | Cells too poor for karyotyping |
| | G.R.O. | F | 22 { 8 | (a) 45–46 | 62 | 46, XX, −7-like, +11-like, −D, +M 1 |
| | | | 14 | (b) 63–70 | 75 | 63, XX, +3 C-like, +4 16-like, +2 17-like, +2 18-like, +2 F-sized, +3 G, +M 1 |
| | O.U.D. | F | 42 (15) { 35 (9) | (a) 44–47 | 71 | 46, XX, −1, +12, +16, −17, −18, −20, +M 1, +M 3 |
| | | | 7 (6) | (b) 71–79 | 57 | 71, XXXXXX, −1, +2, +3, −4, −4, +6, +6, +7, +7, +7, −9, +10, +10, +12, +14, +14, −15, −17, −17, +19, +19, +19, +19, +19, +19, +19, +21, +M 1, +M 2, +M 3, +M 4, +M 5, +M 6, +M 7, +M 8, 6 q− |

karyotype can be given is of course improved for those tumours in which G-banding was possible.

*Modal number.*—The modal number could be unequivocally established in 21 cases (Fig. 1) and the existence of 2 groups is suggested. In the first, the modal number is equal to or very close to 46 (range 43–51) and 11/21 cases were in this group. Cell populations were relatively homogeneous.

In the second group of 9 cases, the mean modal number was close to 65 (range 58–69) and cell populations were slightly more heterogeneous. In one case (N.A.T.), cells were near-tetraploids (modal number 104).

*Double cell populations.*—In 3 cases (G.R.O., O.U.D., H.O.U.), the existence of 2 cell clones was demonstrated by examining the slides. Two modal numbers could be established for each tumour: G.R.O. = 45 and 63, O.U.D. = 46 and 80, H.O.U. = 87 and 160. In one case (H.O.U.), 3/15 cells studied presented an extensive polyploidy centred around 160 chromosomes. The increased chromosome number was primarily at the expense of Groups C and F, but supernumeraries were observed in all the groups. The other 2 cases (G.R.O., O.U.D.) included a population of near-diploid cells: 8/11 cells for G.R.O. and 23/27 for O.U.D. In these 2 cases, numerical and structural anomalies were observed in all the cells examined and primarily involved Group F for G.R.O.

*Supernumerary or missing chromosomes.*—The chromosomal groups affected by supernumerary or missing chromosomes (studied before G-banding) are

FIG. 1.—Distribution of chromosome numbers. ● : modal number and dispersion in each case. The cases studied were ordered in terms of increasing modal number. Only those cases with one cell population are represented on the Fig. (21 cases).

shown in Table II for each case. Supernumerary chromosomes preferentially belonged to Groups C, E and F. In the 22 cases in which a detailed analysis could be performed, supernumeraries were observed in Groups F and C, 17 and 16 times. Missing chromosomes were primarily in Group E and to a lesser extent in Groups A and G. G-banding confirmed the predominance of supernumeraries in Groups C and F and led to the distinction between normal supernumeraries and markers: patient O.U.D. 71.XXXXXXX, −1, +2, +3, −4, −4, +6, +6, 6 q−, +7, +7, +7, −9, +10, +10, +12, +14, +14, −15, −17, −17, +19, +19, +19, +19, +19, +19, +19, +21 and 8 markers.

*Markers and double minutes.*—Markers could be demonstrated in 19/22 cases. Chromosome abnormalities are summarized in Table III. An abnormally long marker, designated # 2 q+, was observed in 6 cases. In 4 of these cases, the tumours belonged to the paradiploid group. Metacentric markers, a bit smaller than F group chromosomes, were demonstrated 6 times, 4 of which were tumors in the para-triploid group. Double-minutes were observed in 5 cases. Their number never exceeded 2/cell and the number of cells involved varied from one case to another. In only one case (A.R.R.) did all the cells carry double-minutes (Fig. 2). The other markers observed were # 1 q+, # 2 q−, # Bp−, #Bq+, dicentric # Cp−, # Dq+ and # Gq−.

*Contribution of G-banding*

This technique furnished details on abnormal chromosomes which had already been located with the standard technique (Fig. 3). Thus, the origin of certain markers could be determined: 1 q−, 6 q−, # 11 q+, $M_3$ = probable isochromosome (O.U.D.), probable iso 17 q (P.O.I.), probable iso 2 p and 2 q− ? (A.B.O.). It should be stressed that, in spite of G-banding, the origin of the marker and its classification remained undetermined in numerous cases. G-band characterization of markers verified their quasi-constancy in several cells of the same patient: one #C (L.A.I.), in the case of O.U.D., the G-banding technique enabled us to demonstrate the existence of a # 11 q+ marker in the 2 cell populations. In certain cases, the existence of a duplication of a # 2 q+ (P.O.I.) and of a #F (L.A.I.) could be affirmed. Concerning the # 2 q+ markers, we observed with the standard technique in 4 cases (P.L.A., P.O.I., H.U.G., L.A.I.) in which G-bands were obtained, that they were different from one case to another. Thus, in one case the marker resulted from the translocation of the short arm of a 2 on to a long arm of undetermined origin (P.O.I.). The origins could not be determined in the other cases (Fig. 4).

The contribution of G-banding was apparently more decisive in the diploid forms. Two of the 3 cases with a modal

TABLE II.—*Supernumerary chromosomes, missing chromosomes in colonic cancer cells (24 cases). The percentage of cells with supernumerary and/or missing chromosomes is indicated for each case.*

| | | | | Supernumerary chromosomes | | | | | | | | | Missing chromosomes | | | | | | | | |
|---|---|---|---|---|---|---|---|---|---|---|---|---|---|---|---|---|---|---|---|---|---|
| | Patient | Sex | Modal number | A | B | C | D | E | F | G | X | Y | A | B | C | D | E | F | G | X | Y |
| Near-diploid | C.H.O. | F | 42–45 | | | 100% | | | | | | | | | | | 66% | 66% | 66% | | |
| | A.B.O. | M | 43–46 | | | | | | 38% | | | | | | | | 15% | | 57% | | |
| | H.O.R. | M | 41–43 | | | | | | | | | | 90% | | | | | | | | |
| | B.R.I. | F | 45–47 | | | poor material for evaluation | | | | | | | | | | | | | | | |
| | H.U.G. | F | 45–47 | 33% | | | | | 44% | | | | | | | | 33% | | | | |
| | P.E.Y. | F | 45–47 | | | | | 22% | 50% | | | | 11% | | | | 33% | | | | |
| | P.L.A. | M | 46–49 | | | | | 77% | 88% | | | | | | 66% | | | | | | |
| | D.A.E. | F | 46–49 | | | 75% | | | 75% | | | | | | | | | | | | |
| | W.E.B. | M | 49–50 | | | 100% | | | | | | | | | | | | | | | |
| | P.O.I. | M | 49–50 | | 62% | 90% | | | 88% | | | | | | | | 87% | | | | |
| | R.I.V. | M | 50–52 | | | 100% | 15% | | | | | | | | | | | 75% | 75% | | |
| Near-triploid | A.R.R. | M | 57–60 | | | 94% | | | 54% | | | | | | | | | | | | |
| | G.U.E. | F | 57–61 | 60% | 50% | 100% | | 80% | 100% | | | | | | | | | | | | |
| | G.E.N. | F | 60–65 | | | | | 100% | 100% | | | | | | | | | | | | |
| | W.E.I. | M | 60–62 | | | 100% | | | 100% | | | | | | | | | | | | |
| | R.O.B. | F | 58–68 | | | 90% | | 90% | 90% | 50% | | | 50% | | | 50% | | | | | |
| | B.E.A. | M | 58–73 | | | poor material for evaluation | | | | | | | | | | | | | | | |
| | L.A.I. | M | 64–67 | | | 100% | | | 60% | | | | | | | | | | | | |
| | L.O.T. | F | 67–69 | | | 80% | | 85% | | | | | | | | | | | | | |
| | C.H.A. | M | 68–70 | | | 100% | 70% | 43% | 100% | | | | | 43% | | | 29% | | 14% | | |
| | N.A.T. | M | 104–106 | 15% | 20% | 100% | 15% | 100% | 100% | | | | | | | | | | | | |
| Two populations | G.R.O. | F | 45–46 | | | | | | | | | | | | | | | | | | |
| | | | 63–70 | | | | | 80% | 80% | 80% | | | | | | | | | | | |
| | H.O.U. | M | 78–92 | 70% | 60% | 100% | 80% | 70% | 100% | 70% | | | | | | | | | | | |
| | | | 159–162 | 100% | 100% | 100% | 100% | 100% | 100% | 100% | | | | | | | | | | | |
| | O.U.D. | F | 44–47 | | | 90% | | 80% | | | | | 90% | | | | 100% | 50% | | | |
| | | | 71–79 | 75% | | 100% | | 75% | 100% | | 75% | | | | | | | | | | |

Table III.—*Common markers observed without banding techniques in 24 colonic cancers*

| | Patient | Sex | Modal number | Marker chromosomes with standard technique classification according to the analogy with different chromosomal groups: A | B | C | D | E | F | G | Minute | Double-minute |
|---|---|---|---|---|---|---|---|---|---|---|---|---|
| Near-diploid | C.H.O. | F | 42–45 | | | | | | | | | |
| | A.B.O. | M | 43–46 | | | | q+ (45%) | | | q− (21%) | | + (22%) |
| | H.O.R. | M | 41–43 | | | | | | | | | |
| | B.R.I. | F | 45–47 | | | | poor material for evaluation | | | | | |
| | H.U.G. | F | 45–47 | # 2 q+ (33%) # 3 sub-medioc (55%) | | | | | smaller (44%) | | + (34%) | |
| | P.E.Y. | F | 45–47 | # 2 q+ (22%) | | | | | | | + (22%) | |
| | P.L.A. | M | 46–49 | | p− (11%) | | q+ (77%) | | | | | |
| | D.A.E. | F | 46–49 | | | p− (25%) | | | | | + (25%) | |
| | W.E.B. | M | 49–50 | | | | | | | | + (16%) | |
| | P.O.I. | M | 49–50 | # 2 q+ (33%) | | p− (14%) | | | | | | |
| | R.I.V. | M | 50–52 | # 2 q+ (60%) | q+ (66%) | | | | | | + (77%) | |
| Near-triploid | A.R.R. | M | 57–60 | # 2 q+ (5%) | dic (2%) | | | | smaller (51%) | | + (37%) | + (75%) |
| | G.U.E. | F | 57–61 | two markers A-size (80%) | | | | | | | | |
| | G.E.N. | F | 60–65 | | | | | | smaller (90%) | | + (39%) | + (15%) |
| | W.E.I. | M | 60–62 | | | | | | | q− (33%) | | |
| | R.O.B. | F | 58–68 | | | | | | smaller (60%) | | + (12%) | |
| | B.E.A. | M | 58–73 | | | | poor material for evaluation | | | | | |
| | L.A.I. | M | 64–67 | | | | | | | | + (42%) | |
| | L.O.T. | F | 67–69 | | | | | | | | + (66%) | |
| | C.H.A. | M | 68–70 | # 2 q+ (50%) | | | | | smaller (70%) | | | + (5%) |
| | N.A.T. | M | 104–106 | Marker larger than chrom 1 (50%) | | | | | smaller (90%) | | + (40%) | |
| Two populations | G.R.O. | F | 45–46 (8 cells) | | | | | | | | | |
| | | | 63–70 (14 cells) | | | | | | | | | |
| | H.O.U. | | 78–92 (12 cells) | | | | | | | | | |
| | | | 159–162 (3 cells) | | | | | | | | + (40%) | + (13%) |
| | O.U.D. | | 44–47 (23 cells) | # 2 q− (90%) | | | | | | | | |
| | | | 71–79 (4 cells) | # 2 q− (50%) | | | | | | | | |

FIG. 2.—Metaphase plate in standard technique (A.R.R.). The arrow indicates a double-minute.

number of 46 (H.U.G., P.E.Y.) were studied with this method. In one of them (H.U.G.) the standard technique demonstrated structural anomalies in A and F chromosomes in all the cells for which a karyotype could be established. G-banding led to the demonstration of a # A, a #2 q+ and a small # F marker, although the origins could not be determined. In another case (P.E.Y.), karyotyping of 7 cells with the standard technique showed that 4 had a normal karyotype, while 3 exhibited numerical and structural anomalies in groups A and F: a # 2 q+, a # 2p−, a Cp− and a supernumerary F. The G-banding technique confirmed structural anomalies without again being able to define the origin of the markers.

*Possible relationships between karyotype and histology, localization, local and metastatic spread, familial cancer and preoperative symptoms*

*Histology.*—We observed 18 adenocarcinomas and 6 mucinous adenocarcinomas. The major cytogenetic characteristics of these 2 types of tumours are indicated in Table IV. The mucinous adenocarcinomas appeared to be preferentially near-diploid or included 2 clones. Most adenocarcinomas were near-triploid or polyploid (11/18 cases). Double-minutes markers were observed uniquely in this group.

*Localization.*—In 3/4 cases of right localization, the tumours were near-diploid (W.E.B., C.H.O., R.I.V.) and near-tetraploid once (N.A.T.). The only case of

FIG. 3.—G-banding karyotype (O.U.D.). Note the large number of marker chromosomes of unknown origin.

FIG. 4.—Examples of several markers observed in 3 cases with the G-banding technique. A.B.O. (a) Short arm of 1 (?) or segmentary duplication of another origin (?)—long arm of unknown origin; (b) probable isochromosome 2p (?); (c) derivative of a short arm of 2 (?). P.O.I. (a) Short arm of 2—long arm of unknown origin; (b) probable isochromosome 17q. W.E.B. Rearranged chromosome 3 (?). Several mechanisms of formation are possible: isochromosome (A.B.O. b/; P.O.I./b), translocation (P.O.I./a), deletion (A.B.O./c), complex rearrangment (W.E.B.).

transverse localization encountered had 46 chromosomes.

*Spreading.*—The cases studied were placed in the 3 classes of Dukes (1932) (Table V). In Class A, the near-diploid forms or those including a near-diploid population were observed 6/8 times. In the 2 other groups combined, this proportion was only 7/16. Double-minutes were observed only in Classes B and C. We noted the presence of small # F markers in Table IV, even though their origin remains undetermined. They were observed only in the invasive cancers of Duke's stage B or C.

*Antecedents with cancers and colonic polyadenoma, symptoms. Relationships with personal cancer antecedents and with the presence of associated colonic polyadenoma on the surgically removed specimen.*—Two of the 3 cases with personal cancer antecedents (R.I.V., H.O.U.) furnished a detailed karyotype analysis. The modal numbers were found to be very different (R.I.V. = 51, H.O.U. = 87 and 160). Independent polyadenomatous lesions associated with the cancer were observed 9 times (Table III).

No relationship was evident from a comparison on the symptomatic and cytogenetic data. It was noted that of the 3 cases (D.A.E., A.R.R., A.B.O.) whose symptoms included long-term fever without peritumoural suppuration, 2 exhibited a double-minute marker.

### DISCUSSION

This study demonstrates the reliability of a method which regularly furnishes the cytogenetic characteristics of primary human colo–rectal carcinomas. In most cases, each cancer was characterized by a single cell clone, although tumours with 2 cell populations were also found. Supernumerary chromosomes belonged primarily to Groups F and C, while missing chromosomes were from Groups E, A and G. The most often observed markers with the standard technique were one # 2 q+, one small metacentric # F and one minute. Double-minutes were observed only in Dukes Stage B and C. Cells were most often paradiploid in non-invasive cancers and structural anomalies were fewer.

The technique presently used is similar to that recommended by Xavier *et al.* (1974). All samples must be taken within 15 min of the surgical specimen being freed and must be taken from the inner face of the peripheral tumoural swelling. In addition, the tissue fragments were distributed in 4–6 culture flasks and it appeared preferable to incubate them for 15–20 h before processing the cells. Colcemid

TABLE IV.—*Comparison between karyotypes and* (1) *histological tumour type* (*adenocarcinoma, mucinous adenocarcinoma*); (2) *localization in colon: ascending RC, transverse TC or descending colon LC;* (3) *site of cancers in antecedents, if any; and* (4) *association with polyadenomas, if any.* (*ORL = oto-rhino-laryngeal*)

| | Patient | Age | Sex | Modal number | dm | Localization | Personal cancer antecedent | Associated colonic polyadenoma |
|---|---|---|---|---|---|---|---|---|
| Adenocarcinoma | A.R.R. | 70 | M | 57–60 | + | LC | | Presence |
| | C.H.A. | 59 | M | 68–70 | + | LC | | |
| | R.I.V. | 71 | M | 50–52 | | RC | ORL | Presence |
| | H.O.U. | 56 | M | 78–92<br>159–162 | + | LC | Colon | |
| | A.B.O. | 52 | M | 43–46 | + | LC | | |
| | P.L.A. | 69 | M | 46–49 | | LC | | |
| | G.E.N. | 56 | F | 60–65 | + | LC | | |
| | B.E.A. | 67 | M | 58–73 | | LC | | Presence |
| | L.O.T. | 78 | F | 67–69 | | LC | | |
| | W.E.I. | 76 | M | 60–62 | | LC | | |
| | B.R.I. | 57 | F | 45–47 | | LC | Breast | Presence |
| | P.O.I. | 53 | M | 49–50 | | LC | | Presence |
| | P.E.Y. | 56 | F | 45–47 | | LC | | |
| | C.H.O. | 73 | F | 42–45 | | RC | | |
| | L.A.I. | 82 | M | 64–67 | | LC | | |
| | N.A.T. | 55 | M | 104–106 | | RC | | Presence |
| | O.U.D. | 73 | F | 44–47<br>71–79 | | LC | | |
| | G.U.E. | 72 | F | 57–61 | | LC | | |
| Mucinous adenocarcinoma | D.A.E. | 79 | F | 46–49 | | LC | | |
| | R.O.B. | 54 | F | 58–68 | | LC | | |
| | H.U.G. | 74 | F | 45–47 | | TC | | |
| | G.R.O. | 83 | F | 45–46<br>63–70 | | LC | | Presence |
| | W.E.B. | 80 | M | 49–50 | | RC | | Presence |
| | H.O.R. | 78 | M | 41–43 | | LC | | Presence |

treatment was reduced, both in incubation time and concentration, in order to prevent excessive chromosome condensation and shortening of metaphases.

Trypsin G-banding (Seabright, 1971), gave interpretable results in only 13 cases, possibly owing to excessive chromosome condensation in certain preparations. We observed, as have others (Sonia, & Sandberg 1978), a general resistance to trypsinization by tumour cells. Despite careful adjustment of trypsin exposure in each case, certain preparations could not be G-banded.

It is expected that G-banding will contribute important findings in diploid forms. Improvement of analytical techniques should be concentrated on demonstrating the earliest chromosomal anomalies. By analogy with the most recent experience in haematology (Yunis, 1981*a*) it may be expected that the G-banding techniques, even the application of high resolution chromosomal analysis to epithelial tissue (Yunis, 1981*b*), will be very useful in the analysis of precancerous lesions and in the chromosomal analysis of the colo–rectal mucosa from subjects with a high risk of neoplasia.

A summary of the most important results obtained until 1979 can be found in the review of Sandberg (1980). The earliest studies had already shown the relatively homogeneous nature of chromosomal anomalies for a given tumour, the existence of 2 distinct groups, near-diploid and near-triploid, and a selection for loss of D and G chromosomes (Atkin & Baker 1969). In the present series, it was Group E especially and chromosomes 17 and 18 in particular, which was affected, Groups A and G being involved to a lesser extent. In a series of 14 cases, Sonia & Sandberg (1978) found that the majority were

TABLE V.—*Relationship between karyotype and local and metastatic spread according to Duke's classification*

| | | Modal number | dm | # F smaller |
|---|---|---|---|---|
| A | G.R.O. | 45–46 | | |
| | | 63–70 | | |
| | B.E.A. | 58–73 | | |
| | B.R.I. | 45–47 | | |
| | W.E.B. | 49–50 | | |
| | P.E.Y. | 45–47 | | |
| | L.A.I. | 64–67 | | |
| | O.U.D. | 44–47 | | |
| | | 71–79 | | |
| | H.O.R. | 41–43 | | |
| B | C.H.A. | 68–70 | + | + |
| | A.B.O. | 43–46 | + | |
| | H.U.G. | 45–47 | | + |
| | G.E.N. | 60–65 | + | + |
| | D.A.E. | 46–49 | | |
| | L.O.T. | 67–69 | | |
| | P.O.I. | 49–50 | | |
| | N.A.T. | 104–106 | | |
| C | H.O.U. | 78–92 | + | |
| | | 159–162 | | |
| | A.R.R. | 57–60 | + | + |
| | R.I.V. | 50–52 | | |
| | P.L.A. | 46–49 | | |
| | R.O.B. | 58–68 | | + |
| | W.E.I. | 60–62 | | |
| | C.H.O. | 42–45 | | |
| | G.U.E. | 57–61 | | |

polyploid. Supernumerary chromosomes were observed mostly in Group D but also in Groups C, E and G, while missing chromosomes involved Group C and to a lesser extent Groups B and F. Markers were rare, but were observed more often in cancers which metastasized. In our series, the numbers of near-diploid and near-triploid tumours were about the same. A novel finding is the coexistence of 2 abnormal cell lines in 3 cases. The comparison of modal number of these cases with the degree of spread of the cancer generates several concepts on the clonal evolution of colo–rectal cancers. In 2 Class A cases, modal numbers were 45/63 (G.R.O.), and 45/78 (O.U.D.), while for the other case, Class C, it was 87/160 (H.O.U.). The cases could correspond to transition phases from one clone to another, in agreement with the hypothesis of Atkin (1976).

The existence of a double population could also be a stable characteristic of the cancer. This hypothesis is supported by the recent work of Dexter *et al.* (1981), who demonstrated the existence of 2 well defined clones in the same adenocarcinoma of the sigmoid colon, based on cytogenetics, histology and chemosensitivity.

Martin *et al.* (1979) utilized L-arterenol in order to obtain a larger number of analysable metaphases. In spite of this, they were able to determine chromosome numbers only in 13/17 cases. Detailed karyotyping could only be performed 6 times. Although we encountered the same difficulties, it appears that the present method has a higher yield. The number and type of markers described by the above authors are similar to those we observed, suggesting that primary metastatic tumours were sampled. The presence of a # 2 q+ marker was observed in a hypodiploid tumour. In the present study we identified this marker 6 times.

Recent results by Reichmann *et al.* (1980, 1981) stressed 2 essential points of chromosome analysis of solid tumours: (i) the relation between the early acquired chromosome aberrations and the cancer itself and (ii) the presence of double-minutes. We agree with these authors that much attention should be paid to cancers with a diploid line. Three of our cases had clones with 46 chromosomes and 2 of them could be analysed in detail. Both had a #2q+ marker and one minute in common.

It is consistent with these observations that 2 processes could occur in the early developmental stages: (i) the very limited appearance of structural and/or numerical anomalies primarily involving groups A and F would subsequently lead to near-diploid cancers; (ii) the appearance of a diploid/hyperdiploid mosaic would subsequently lead to near-triploid cancers.

Double-minutes markers were recently reported for the first time in 2 cases of human colo–rectal cancers (Reichmann *et al.*, 1980). The number of double-minutes per cell and the number of cells involved were both highly variable. were We able to demonstrate double-minutes in 5 cases in the present study, each an invasive form (Duke's stage B and C).

Chromosomal fragments and double-minutes are to be distinguished by the following criteria: presence or absence/cell (constant or inconstant ratio), number/cell, constant or variable size (Levan & Levan, 1978; Barker & Hsu, 1978). The nature of double-minutes remains to be elucidated. The absence of a centromere explains the irregular distribution of this marker in daughter cells during mitosis. The double-minutes we observed did not arise as artefacts or from associated therapies, since culture *in vitro* never exceeded 24 h and none of the patients received preoperative chemo- or radio-therapy. The demonstration of double-minutes could be prognostically important, since the maintenance or even the amplification of these structures with cell divisions suggest that they confer a selective advantage on the cell bearing them (Trent, 1980). In addition, methotrexate sensitivity of cultured malignant cells is related to the level of dihydrofolate reductase, the increased level of which induces drug resistance. The genetic stuctures responsible for this have been identified as the homogeneously staining regions (HSR), related to a stable methotrexate resistance. Double-minutes confer variable resistance as a function of their quantitative importance (Alt *et al.*, 1978; Bostock *et al.*, 1979; Kaufman *et al.*, 1979). In addition to their theoretical interest, these results open interesting perspectives on the possible relationships between the cytogenetic examination of a tumour and its chemo-sensitivity.

The initial results with colonic cancer suggest that the survival time of near-triploid forms is greater than that of near-diploid (Martin *et al.*, 1979). As a result of insufficient follow-up times, we could not substantiate these arguments. Nevertheless, available data concerning malignant haemopathies (Golomb & Rowley, 1981) provide the motivation for carefully following this problem in the context of malignant tumours of the digestive tract.

This investigation was supported in part by a grant from UER Cochin Port-Royal (contract No. 5281R) and in part by a grant from the Comité Parisien de la Ligue Nationale Française contre le cancer.

The authors thank Dr R. Berger for his assistance in the interpretation of certain karyotypes.

### REFERENCES

ALT, F., LELLEMS, R., BERTINO, J. & SCHIMKE, R. (1978) Selective multiplication of dihydrofolate reductase genes in methotrexate-resistant variants of cultured murine cells. *J. Biol. Chem.*, **253**, 1357.

ATKIN, N. B. (1976) Cytogenetic aspects of malignant transformation. In *Experimental Biology and Medicine, Monographs on Interdisciplinary topics*, Vol. 6, Basel: S. Karger.

ATKIN, N. B. & BAKER, M. C. (1969) Possible differences between the karyotypes of preinvasive lesions and malignant tumours. *Br. J. Cancer*, **23**, 329.

BARKER, P. E. & HSU, T. C. (1978) Are double minutes chrosomes? *Exp. Cell. Res.*, **113**, 457.

BOSTOCK, C. J., CLARK, E. M., HARDING, N. G. L. & 4 others (1979) The development of resistance to methotrexate in a mouse melanoma cell line. *Chromosoma*, **74**, 153.

DANES, B. S. (1978) Increased *in vitro* tetraploidy: tissue specific within the heritable colorectal cancer syndromes with polyposis coli. *Cancer*, **41**, 2330.

DEXTER, D. L., SPEMULLI, E. H., FLIGIEL, Z. & 4 others (1981) Heterogeneity of cancer cells from a single human colon carcinoma. *Am. J. Med.*, **71**, 949.

DUKES, C. E. (1932) The classification of cancer of the rectum. *J. Pathol.*, **35**, 323.

ENTERLINE, H. T. & ARVAN, D. A. (1966) Chromosome constitution of adenoma and adenocarcinoma of the colon. *Cancer*, **20**, 1746.

GOLOMB, H. M. & ROWLEY, J. D. (1981) Significance of cytogenetic abnormalities in acute leukemias. *Hum. Pathol.* **12**, 515.

GRANBERG, I., GUPTA, S. & ZECH, L. (1973) Chromosome analyses of a metastatic gastric carcinoma including quinacrine fluorescence. *Hereditas*, **75**, 189.

ISCN (1978) *Cytogenet & Cell Genet.*, **21**, 311.

JACKSON, J. F. (1967) Chrosome analysis of cells in effusions from cancer patients. *Cancer*, **20**, 537.

KAUFMAN, R. J., BROWN, P. C. & SCHIMKE, R. T. (1979) Amplified dihydrofolate reductase genes in unstably methotrexate-resistant cells are associated with double minute chromosomes. *Proc. Natl Acad. Sci.*, **76**, 5669.

LABOISSE, C. L., AUGERON, C., COUTURIER-TURPIN, M. H., GESPACH, C., CHERET, A. M. & POTET, F. (1982) Characterization of a newly established human gastric cancer cell line bearing histamine $H_2$ receptors: HGT-1. *Cancer Res.*, **42**, 1541.

LEIBOVITZ, A., STINSON, J. C., McCOMBS, W. B., McCOY, C. E., MAZUR, K. C. & MABRY, N. D. (1976) Classification of human colo-rectal adenocarcinoma cell line. *Cancer Res.*, **36**, 4562.

LEVAN, A. & LEVAN, G. (1978) Have double-minutes functioning centromeres? *Hereditas*, **88**, 81.

LUBS, H. A. & CLARK, R. (1963) The chromosome complement of human solid tumors: I. Gastrointestinal tumors and technic. *N. Engl. J. Med.*, **268**, 907.

MAKINO, S., SASAKI, M. S. & TONOMURA, A. (1964) Cytological studies of tumors XL chromosome studies in fifty-two human tumours. *J. Natl Cancer Inst.*, **32**, 741.

MARTIN, P., LEVIN, B., GOLOMB, H. M. & RIDDELL, R. H. (1979) Chromosome analysis of primary large bowel tumors: a new method to improving the yield of analyzable metaphases. *Cancer*, **44**, 1656.

MITELMAN, F., LEVAN, G. (1978) Clustering of aberrations to specific chromosomes in human neoplasms. III. Incidence and geographic distribution of chromosome aberrations in 856 cases. *Hereditas*, **89**, 207.

MORSON, B. C. (1976) Types histologiques des tumeurs intestinales. *Classification Histologique Internationale des Tumeurs*, Geneve: OMS. p. 23.

PARIS CONFERENCE (1971) Standardisation in human cytogenetics. In *Birth Defects, Original Article Series* (Ed. Bergsma) Vol. 8, White Plains, N.Y.: National Foundation, March of Dimes, 1972.

PARIS CONFERENCE (1971) Supplement (1975): Standardisation in human cytogenetics. In *Birth Defects, Original Article Series*, (Ed. Bergsma) Vol. 11, White Plains, N.Y.: National Foundation, March of Dimes,.

REEVES, B. R. & HOUGHTON, J. A. (1978) Serial cytogenetic studies of human colonic tumour xenografts. *Br. J. Cancer*, **37**, 612.

REICHMANN, A., RIDDELL, R. H., MARTIN, P. & LEVIN, B. (1980) Double-minutes in human large bowel cancer. *Gastroenterology*, **79**, 334.

REICHMANN, A., MARTIN, P. & LEVIN, B. (1981) Chromosomal banding patterns in human large bowel cancer. *Int. J. Cancer*, **28**, 431.

SANDBERG, A. A., ISHIHARA, T., MOORE, G. E. & PICKREN, J. W. (1963) Unusually high polyploidy in a human cancer. *Cancer*, **16**, 1246.

SANDBERG, A. A. (1980) *The Chromosomes in Human Cancer and Leukemia*. New York: Elsevier.

SEABRIGHT, M. (1971) A rapid banding technique for human chromosomes. *Lancet*, **ii**, 971.

SONIA, S. I. & SANDBERG, A. A. (1978) Chromosomes and causation of human cancer xxx Banding studies of primary intestinal tumors. *Cancer*, **41**, 164.

TRENT, J. M. (1980) Probing the genetic complement of cancer: possible roles for double minutes in malignancy. *Gastroenterology*, **79**, 392.

TRENT, J. M., SALMON, S. E. (1980) Human tumour karyology: marked analytic improvement by short-term agar culture. *Br. J. Cancer*, **41**, 867.

XAVIER, R. G., PROLLA, J. C., BEMVENUTI, G. A. & KRISMER, J. B. (1974) Tissue cytogenetic studies in chronic ulcerative colitis and carcinoma of the colon. *Cancer*, **34**, 684.

YAMADA, K. & SANDBERG, A. A. (1966) Preliminary notes on the chromosomes of eleven primary tumors of colon. *Proc. Jap. Acad.*, **42**, 168.

YUNIS, J, J. (1981*a*) Specific fine chromosomal defects in cancer: an overview. *Hum. Pathol.*, **12**, 503.

YUNIS, J. J. (1981*b*) New chrosomome techniques in the study of human neoplasia. *Hum. Pathol.* **12**, 540.

YUNIS, J. J., BLOOMFIELD, C. D. & ENSRUD, K. (1981) All patients with acute non lymphocytic leukemia may have a chromosomal defect. *N. Engl. J. Med.*, **305**, 135.